# Evaluation of the relationship between periodontal bone destruction and mesial root concavity of the maxillary first premolar

**DOI:** 10.1186/s12903-024-04494-1

**Published:** 2024-06-26

**Authors:** Zehra Beycioglu, Buket Acar, Mert Ocak, Ibrahim Sevki Bayrakdar, Guliz N. Guncu, Abdullah C. Akman

**Affiliations:** 1 Department of Periodontology, Faculty of Dentistry, Hacettepe University, Ankara, Turkey; 2 Anatomy, Department of Basic Medical Sciences, Faculty of Dentistry, Ankara University, Ankara, Turkey; 3 Department of Oral and Maxillofacial Radiology, Eskişehir Osmangazi University, Eskişehir, Turkey

**Keywords:** Cone beam computed tomography, Periodontitis, Premolar, Risk factors, Tooth root

## Abstract

**Background:**

The purpose of this study was to investigate the morphology of maxillary first premolar mesial root concavity and to analyse its relation to periodontal bone loss (BL) using cone beam computed tomography (CBCT) and panoramic radiographs.

**Methods:**

The mesial root concavity of maxillary premolar teeth was analysed via CBCT. The sex and age of the patients, starting position and depth of the root concavity, apicocoronal length of the concavity on the crown or root starting from the cementoenamel junction (CEJ), total apicocoronal length of the concavity, amount of bone loss both in CBCT images and panoramic radiographs, location of the furcation, length of the buccal and palatinal roots, and buccopalatinal cervical root width were measured.

**Results:**

A total of 610 patients’ CBCT images were examined, and 100 were included in the study. The total number of upper premolar teeth was 200. The patients were aged between 18 and 65 years, with a mean age of 45.21 ± 13.13 years. All the teeth in the study presented mesial root concavity (100%, *n* = 200). The starting point of concavity was mostly on the cervical third of the root (58.5%). The mean depth and buccolingual length measurements were 0.96 mm and 4.32 mm, respectively. Depth was significantly related to the amount of alveolar bone loss (F = 5.834, *p* = 0.001). The highest average concavity depth was 1.29 mm in the group with 50% bone loss. The data indicated a significant relationship between the location of the furcation and bone loss (X^2^ = 25.215, *p* = 0.003). Bone loss exceeded 50% in 100% of patients in whom the furcation was in the cervical third and in only 9.5% of patients in whom the furcation was in the apical third (*p* = 0.003).

**Conclusions:**

According to the results of this study, the depth of the mesial root concavity and the coronal position of the furcation may increase the amount of alveolar bone loss. Clinicians should be aware of these anatomical factors to ensure accurate treatment planning and successful patient management.

## Background

Periodontal diseases encompass a spectrum of inflammatory conditions that affect the supportive structures of teeth, leading to consequential attachment and bone loss and ultimately causing spontaneous tooth loss or necessitating extraction [1]. The microbial biofilm that forms on the tooth surface contributes to chronic inflammation, exhibiting both localized and systemic destruction. An increasing body of evidence emphasizes the reciprocal relationship between an individual’s periodontal health and systemic well-being, implying the importance of periodontal disease prevention and treatment strategies. Periodontitis can manifest in a variety of ways, including localized or generalized presentations or only in the molar-incisor regions. An increase in the severity and rate of progression of the disease may be observed due to the influence of environmental, systemic, and local risk factors such as the anatomy of the roots [2, 3]. A narrow furcation entrance, root concavity, enamel pearl, cervical enamel extensions, root length and width, interroot angle, and root body length are all notable local anatomical factors that may increase the risk of periodontal attachment loss. Therefore, these factors must be taken into consideration when determining patient prognosis, diagnosis, and treatment [4].

Upper first premolar teeth have different root morphologies than other premolar teeth. This is characterized by various configurations and shapes throughout the dentition. Commonly identified anatomical features include bifurcated roots, narrow furcation entrances, multiple canals, and deep mesial concavities [5]. Concavities can be found on the furcation roof, either coronal or apical to the furcation, as well as on interproximal root surfaces. Identifying these concavities is a diagnostic challenge that frequently necessitates patient anaesthesia during nonsurgical therapy or surgical root exposure. As with any anatomic feature, their presence can affect the progression of attachment loss by harbouring bacterial plaque, making the removal of subgingival calculus and root planning more challenging [6].

Radiographic visualization of periodontal bone loss, subgingival calculus, periapical lesions, and the periodontal gap is critical for effective dental and periodontal treatment. The prevailing method for radiographic examination of periodontal structures involves two-dimensional (2D) imaging modalities, such as intraoral images and extraoral panoramic radiographs, which have inherent limitations caused by superpositions [7]. Projection geometry makes image interpretation difficult and therefore requires clinical experience. Even experienced dentists may fail to detect dental and periodontal pathologies during a radiological examination [8]. CBCT images, which can eliminate the limitations of 2D radiographs, are now widely used to examine both the root and the anatomical structures surrounding it [9]. CBCT provides a more accurate analysis of periodontal defect morphology than conventional clinical and two-dimensional radiographic measurements [10].

Zhao et al. investigated the relationship between upper premolar root concavity and periodontal disease and discovered that the presence of different types of root concavity in the first premolars was associated with both clinical indices of chronic periodontitis and the presence of alveolar bone defects [11]. With an impact on disease progression, concavities can also compromise a patient’s oral health care and interfere with the accessibility of adequate subgingival scaling, which can lead to unresponsiveness to treatment [12].

The null hypothesis for the present study predicted that the localization and morphological characteristics of the upper premolar root concavities may have no impact on the severity of periodontal destruction. The present study was aimed to investigate the morphology of maxillary first premolars’ mesial root concavity and to analyze periodontal bone loss using CBCT and panoramic radiographs and to evaluate their relations.

## Methods

### Study design

The study protocol of this retrospective study was approved by the Health Sciences Research Ethics Committee of Hacettepe University (RESEARCH NO: SBA 23/315). To maintain patient confidentiality, only sex and age were recorded.

### Study population

The source of data used in the present study was the digital oral radiology archive of the Department of Periodontology Faculty of Dentistry, Hacettepe University. All CBCT and panoramic radiographs obtained between January 2015 and November 2023 were included in this study. The inclusion criteria were having both upper first premolars and canine teeth bilaterally, being between the ages of 18 and 65 years, and having previously undergone both CBCT and panoramic radiographs with adequate image quality. Samples with any restorations or deep caries on the mesial side of the first premolar tooth that may make the analysis difficult and those with open contacts > 1 mm were excluded. A total of 610 CBCT images were examined. From these CBCTs, the study was conducted with CBCT images of 100 patients who met the inclusion criteria. This number was determined according to power analysis which was explained in a detailed way in statistical analysis part. Maxillary right and left first premolar teeth were evaluated in each CBCT image, and consequently this study was carried out with 100 right upper and 100 left upper first premolar teeth.

### CBCT image acquisition parameters and image processing

CBCT images were obtained using an i-CAT Next Generation device (Imaging Sciences International, Hatfield, USA) with a tube voltage of 120 kVp, a tube current of 5 mA and a voxel size of 0.2 mm. For all the samples, the slice thickness was 0.2 mm. The images were obtained from two panoramic X-ray units (Orthophos XG 5; Sirona Dental Systems GmbH, Bensheim, Germany, and Veraview IC5 HD; J. Morita Manufacturing Corp., Kyoto, Japan). Voltage values of both devices were between 60 and 70 kVp and current values were between 1 and 7.5 mA. The images were transferred to a digital archiving system (Extreme Pacs, Ankara, Turkey). Images with unsuitable image quality and resolution were excluded from the study. Since all the measurements and analysis were performed by the same researcher (ZB), inter-examiner calibration test was not performed. Intra-examiner reliability was estimated using by the intraclass correlation coefficient (ICC) value, and accordingly, the repeatability and consistency of the measurements were found to be very high (98%).

A researcher (ZB) measured the following parameters using the I-cat software and the data was recorded in Microsoft Excel® (Microsoft Inc., WA, USA):

- Location where the concavity begins (root or crown).
- Depth of the concavity.
- Buccolingual length of the concavity.
- Apicocoronal length of the concavity on the crown starting from the CEJ.
- Apicocoronal length of the concavity on the root starting from the CEJ.
- Total apical length of the concavity.
- Amount of bone loss both by CBCT and panoramic radiography.
- Location of the furcation.
- Length of the buccal and palatinal roots.
- Cervical root thickness buccopalatinally.

**Fig. 1:**
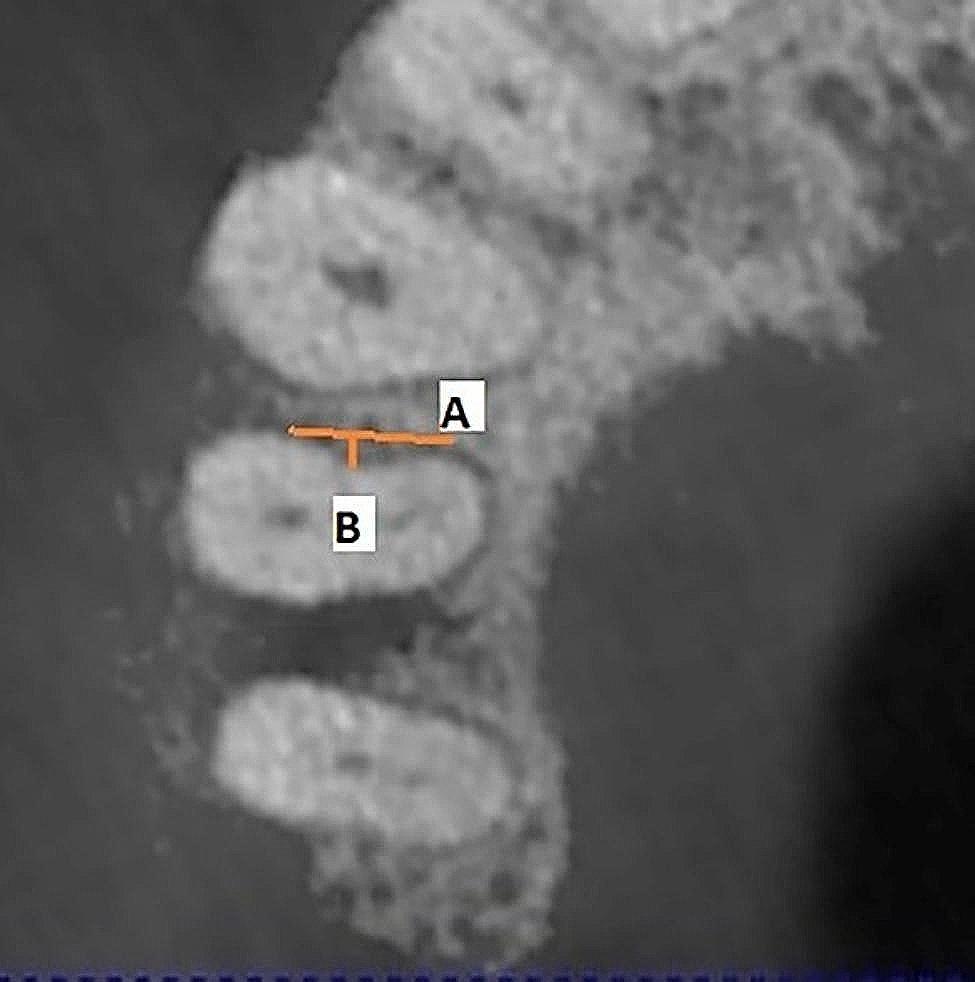
Measuring of depth and buccopalatinal length of the concavity. Line **A**: the distance between the beginning and end of the concavity in the buccopalatinal direction, Line **B**: depth of the concavity. The length of line B was measured by drawing perpendicular to line A from the point where the concavity is deepest

**Fig. 2:**
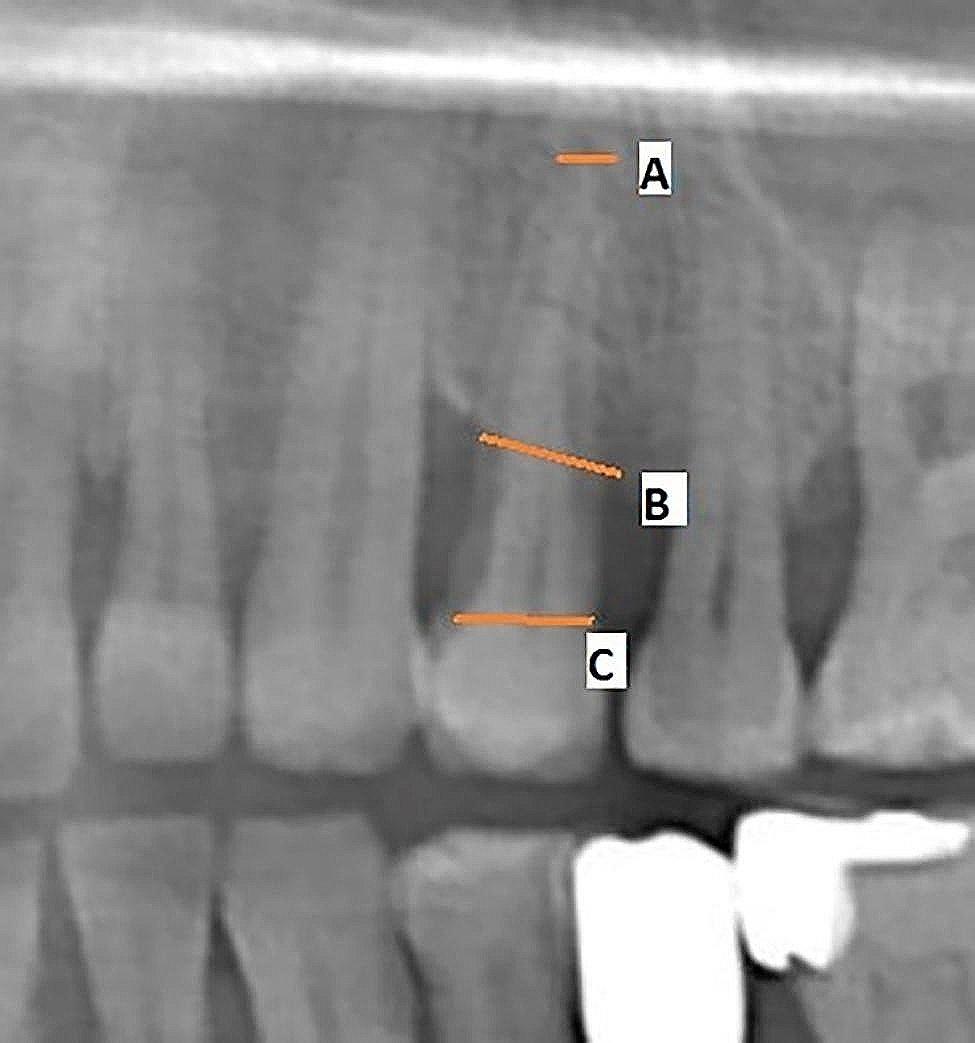
Measuring bone loss on a panoramic radiograph. **A**: apex of the root, **B**: bone level, **C**: cemento-enamel junction. AC/CB shows the rate of bone loss

**Fig. 3:**
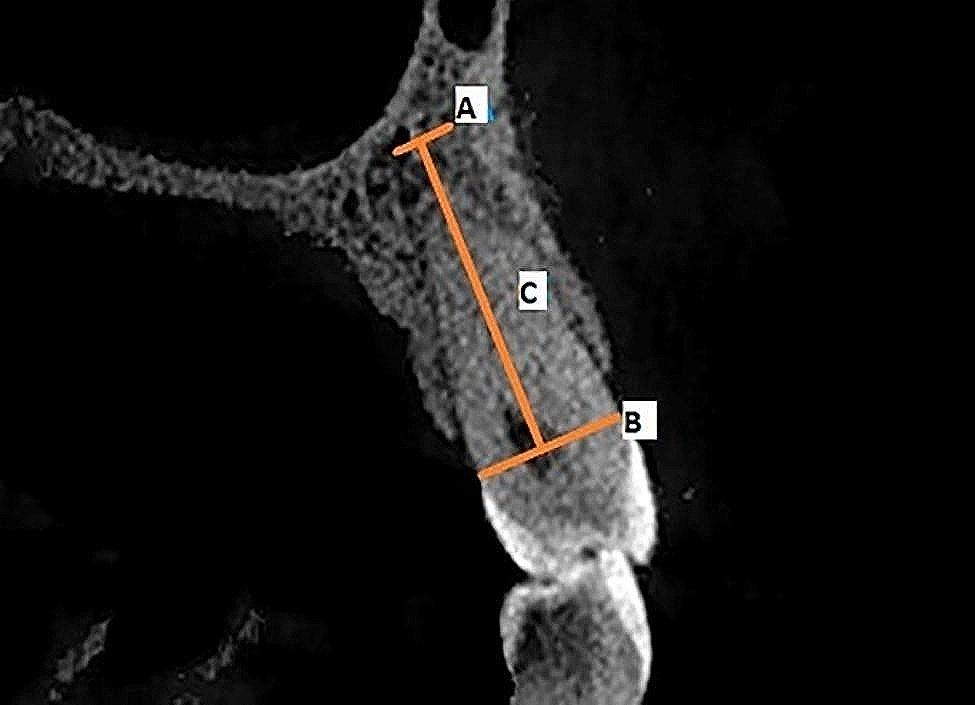
Root length measurements. **A**: apex of the root, **B**: cemento-enamel junction, **C**: root length

**Fig. 4:**
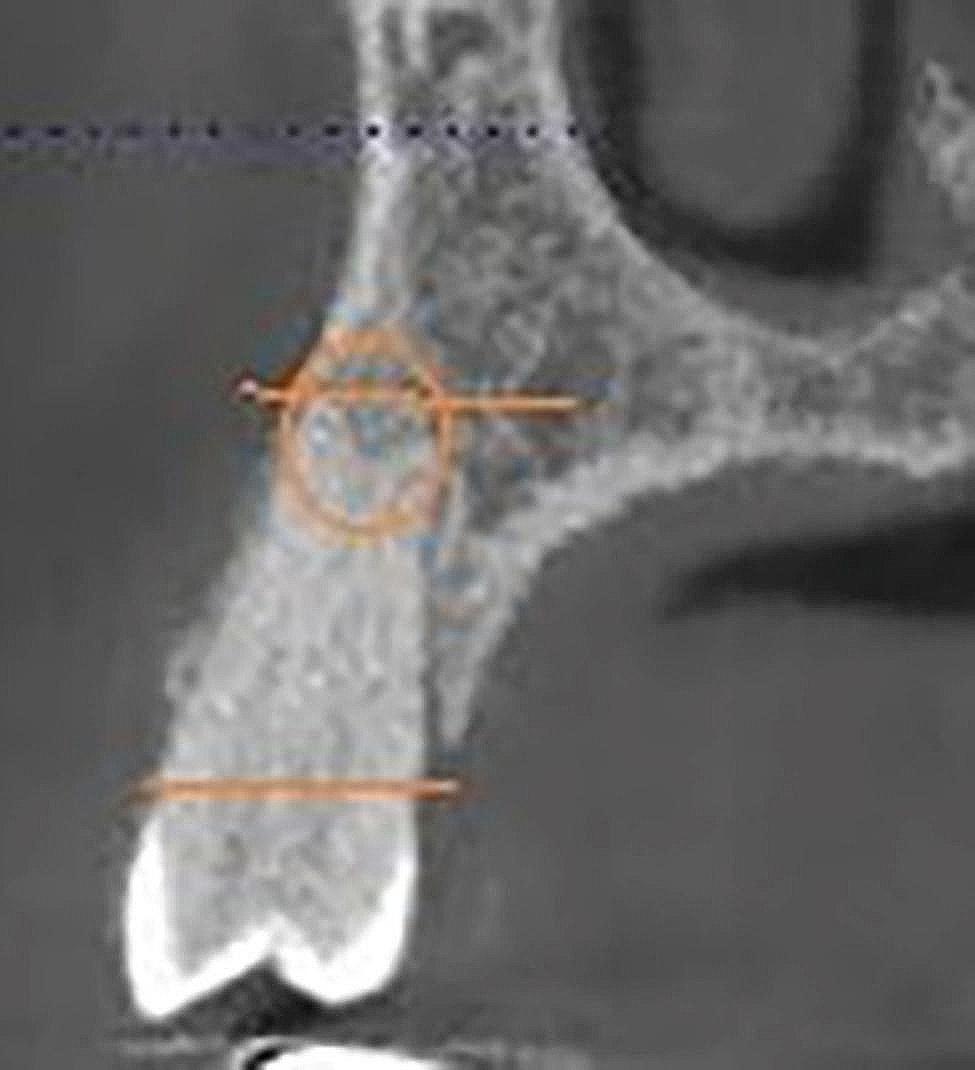
Apically located furcation

**Fig. 5:**
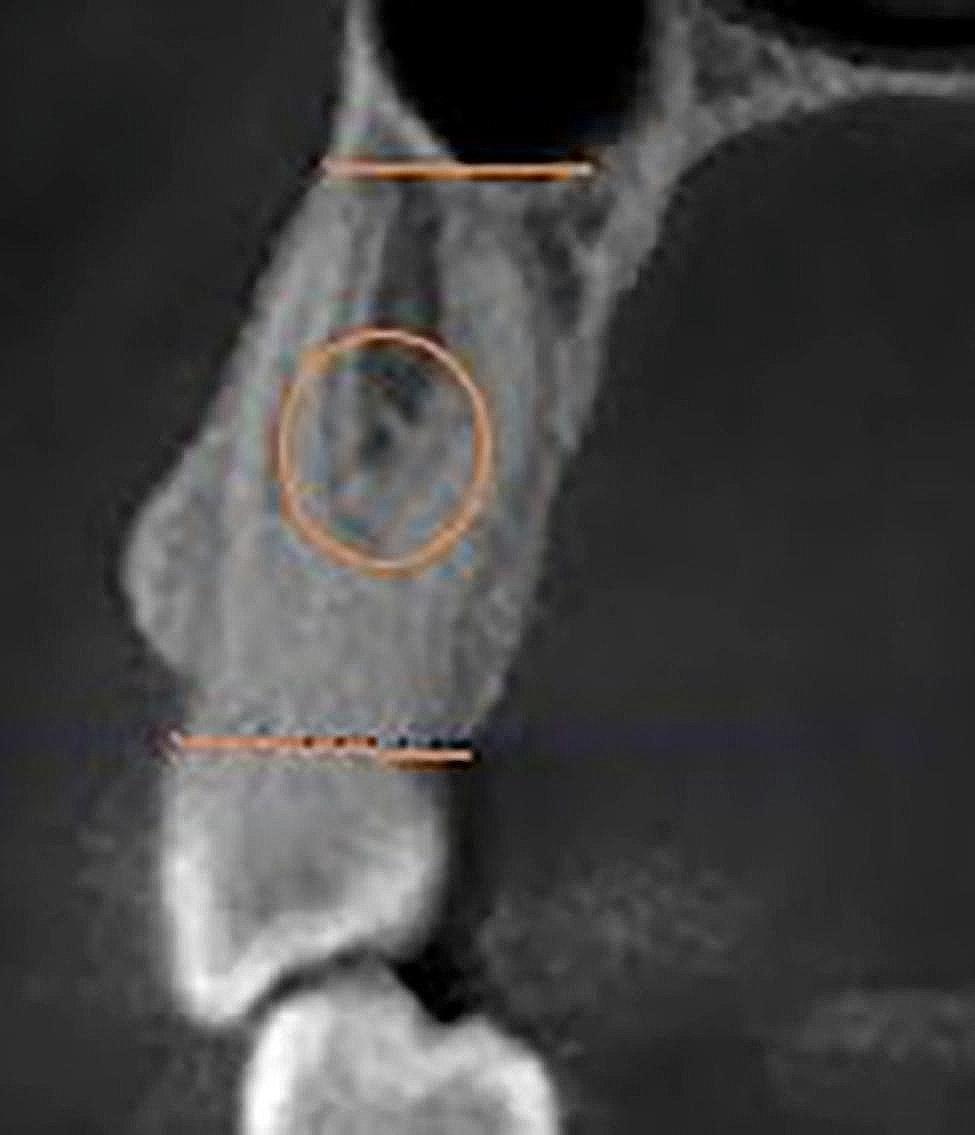
Furcation located in the middle third of the root

Figures 1,  2, 3, 4 and 5 illustrate the measurement references for the morphology of the roots.

### Statistical analysis

Specific software (G*Power 3.1) was utilized to calculate the sample size based on data from a previous study [11]. According to the power analysis, when the effect size was 0.86, the sample size was determined to be *N* = 62 with α = 0.05 and 95% power. However, considering the possibility of missing data, the study was planned to be conducted with a larger population than expected, with *N* = 100 for each group.

For the data analysis, the tests were performed with SPSS (v.24, IBM Corp., NY, USA). Cross tables have been used to examine the relationships and distributions between variables. The chi-square test was applied to evaluate the significance of the relationships of categorical variables such as sex, presence of concavity and starting point, amount of bone loss, furcation location and number of roots with the amount of bone loss. ANOVA was used to examine the relationships of age, depth, and other continuous measures with the amount of bone loss. This test enabled comparison of mean values between different bone loss groups. *p* < 0.05 indicated a statistically significant difference.

## Results

CBCTs from 610 patients were examined, 100 of whom were included in the study, and the total number of upper premolar teeth was 200 (100 on the right side and 100 on the left side).

Table 1 summarizes the age and sex of the patients. This study included 100 individuals aged between 18 and 65 years, with a mean age of 45.21 years (SD = 13.13, Median = 46.50). The sex distribution of the participants revealed that females constituted the majority of the sample (59%), while males represented 41%.

**Table 1:** Demographic data of the participants

		Minimum	Maximum	Mean	SD	Median	*n*	%
**Age**		18	65	45.21	13.13	46.5		
**Sex**	Female						59	59
	Male						41	41

SD, standard deviation; *n*, number

Table 2 shows the measurements/assessments of root morphology and alveolar bone loss. All the teeth in the study had mesial root concavity (100%, *n* = 200). The starting point of concavity was mostly on the cervical third of the root (58.5%). The average depth and buccolingual length measurements were 0.96 ± 0.37 mm (Median = 1.00) and 4.32 ± 0.89 mm (Median = 4.23), respectively. The average distances from the CEJ on the crown and on the root were 0.54 ± 0.82 mm and 7.21 ± 2.06 mm, respectively.

No bone loss was observed in 46% of the samples, while 24.5% of the teeth had less than 25%, 20.5% of the teeth had 25–50%, and 9% of the teeth had more than 50% BL. Based on panoramic X-ray results, bone loss rates were similarly distributed: 49.5% had no bone loss, 23% had less than 25%, 20% had between 25 and 50%, and 7.5% had more than 50% BL.

The data showed that 46.5% of the teeth had no furcation, while the remaining teeth had furcation mostly in the apical third (31.5%). Regarding the number of roots, 46.5% of the samples were single-rooted, and 53.5% were double-rooted.

The average buccal and palatal root lengths were 13.45 ± 1.71 mm and 12.56 ± 1.71 mm, respectively. The mean buccopalatal cervical root width was 8.23 ± 0.73 mm. Tables 3 and 4 show the measurements of the upper right and left premolar teeth, respectively.

The results of the chi-square test and cross-tabulation are presented in Table 5, a complex table. The associations between the amount of bone loss and several variables (sex, tooth number, concavity and starting point presence, amount of bone loss on panoramic X-ray, furcation position, and number of roots) are shown in this table.

The results of the chi-square test indicated a significant relationship between sex and bone loss (X^2^ = 12.242, *p* = 0.007). Male subjects experienced no bone loss at a rate of 32.9%, compared to 55.1% of females. Moreover, males experienced higher rates of bone loss of over 50% (14.6%) compared to female subjects (5.1%).

**Table 2:** Features of the Root Morphologies and Alveolar Bone Loss Measurements

		*n*	%	Minimum	Maximum	Mean	SD	M
**Presence of concavity**	No	0	0					
	Yes	200	100					
**Origin of the concavity**	Cervical third of the crown	71	35.5					
	Cervical third of the root	117	58.5					
	Middle third of the root	7	3.5					
	Apical third of the root	0	0					
	Middle third of the crown	5	2.5					
**Depth of the concavity (mm)**				0.2	2.09	0.96	0.37	1
**Buccolingual length (mm)**				1.71	6.4	4.32	0.89	4.23
**Distance from CEJ on the crown (mm)**				0	3.9	0.54	0.82	0
**Distance from CEJ on the root (mm)**				1.22	12.46	7.21	2.06	7
**Apicocoronal length (mm)**				1.22	14.69	7.75	2.25	7.8
**Amount of bone loss (BL) (CBCT)**	0	92	46					
	< 25%	49	24.5					
	25-50%	41	20.5					
	> 50%	18	9					
**Amount of bone loss (BL) (Panoramic radiograph)**	0	99	49.5					
	< 25%	46	23					
	25-50%	40	20					
	> 50%	15	7.5					
**Location of furcation**	0	93	46.5					
	Cervical Third	2	1					
	Middle Third	42	21					
	Apical Third	63	31.5					
**Number of roots**	Single Root	93	46.5					
	Double Root	107	53.5					
**Buccal root length (mm)**				7.22	17.6	13.45	1.71	13.61
**Palatinal root length (mm)**				7.77	15.88	12.56	1.71	12.61
**Buccopalatinally cervical root width (mm)**				6.2	9.8	8.23	0.73	8.4

*n*, number; SD, standard deviation; M, median

**Table 3:** Measurements of the upper right premolars

		*n*	%	Minimum	Maximum	X	SD	M
**Presence of concavity**	No	0	0					
	Yes	100	100					
**Origin of the concavity**	Cervical third of the crown	37	37					
	Cervical third of the root	60	60					
	Middle third of the root	2	2					
	Apical third of the root	0	0					
	Middle third of the crown	1	1					
**Depth of the concavity (mm)**				0.2	2.09	0.99	0.36	1
**Buccolingual length (mm)**				1.81	6.25	4.27	0.88	4.22
**Distance from CEJ on the crown (mm)**				0	3.4	0.52	0.75	0
**Distance from CEJ on the root (mm)**				1.22	12.46	7.16	2.11	7
**Apicocoronal length (mm)**				1.22	12.46	7.68	2.27	7.51
**Amount of bone loss (BL) (CBCT)**	0	48	48					
	< 25%	22	22					
	25–50%	21	21					
	> 50%	9	9					
**Amount of bone loss (BL) (Panoramic radiograph)**	0	50	50					
	< 25%	23	23					
	25–50%	19	19					
	> 50%	8	8					
**Location of furcation**	0	41	41					
	Cervical Third	1	1					
	Middle Third	20	20					
	Apical Third	38	38					
**Number of roots**	Single Root	41	41					
	Double Root	59	59					
**Buccal root length (mm)**				7.22	17.6	13.31	1.73	13.62
**Palatinal root length (mm)**				7.77	15.42	12.33	1.63	12.6
**Buccopalatinally cervical root width (mm)**				6.2	9.8	8.17	0.77	8.24

n, number; SD, standard deviation; M, median

**Table 4:** Measurements of the upper left premolars

		*n*	%	Minimum	Maximum	Mean	SD	M
**Presence of concavity**	Absent	0	0					
	Present	100	100					
**Origin of the concavity**	Cervical third of the crown	34	34					
	Cervical third of the root	57	57					
	Middle third of the root	5	5					
	Apical third of the root	0	0					
	Middle third of the crown	4	4					
**Depth of the concavity (mm)**				0.2	2.01	0.94	0.38	0.95
**Buccolingual length (mm)**				1.71	6.4	4.37	0.91	4.35
**Distance from CEJ on the crown (mm)**				0	3.9	0.57	0.89	0
**Distance from CEJ on the root (mm)**				2.21	11.6	7.25	2.02	7.44
**Apicocoronal length (mm)**				2.21	14.69	7.82	2.25	8
**Amount of bone loss (BL) (CBCT)**	0	44	44					
	< 25%	27	27					
	25–50%	20	20					
	> 50%	9	9					
**Amount of bone loss (BL) (Panoramic radiograph)**	0	49	49					
	< 25%	23	23					
	25–50%	21	21					
	> 50%	7	7					
**Location of furcation**	0	52	52					
	Cervical Third	1	1					
	Middle Third	22	22					
	Apical Third	25	25					
**Number of roots**	Single Root	52	52					
	Double Root	48	48					
**Buccal root length (mm)**				8.41	16.8	13.6	1.69	13.61
**Palatinal root length (mm)**				9.07	15.88	12.85	1.78	12.91
**Buccopalatinally cervical root width (mm)**				6.4	9.7	8.29	0.7	8.4

*n*, number; SD, standard deviation; M, median

Bone loss was similar between the right and left premolar teeth (X^2^ = 0.709, *p* = 0.871). Both teeth showed comparable distributions of bone loss.

The presence of concavity and the starting point of concavity did not have a statistically significant impact on bone loss. (X^2^ = 7.950, *p* = 0.539). Bone loss was greater in teeth where concavity started at the root cervical third and crown cervical third.

A significant association (X^2^ = 325.381, *p* = 0.000) was observed between the amount of bone loss on panoramic X-ray and CBCT images. Panoramic X-ray revealed a comparable significant loss in 86.7% of patients with more than 50% bone loss compared to 89.9% of those with no bone loss who had bone loss on CBCT. However, panoramic radiographs revealed underdiagnosis of early-stage bone loss and severe bone loss in almost 1 in 10 patients.

The data indicated a significant relationship between furcation site and bone loss (X^2^ = 25.215, *p* = 0.003). In 100% of the patients with furcation in the cervical third and 9.5% in the apical third, there was more than 50% bone loss.

There was no discernible relationship between the number of roots and bone loss (X^2^ = 4.185, *p* = 0.242). The distributions of bone loss rates in teeth with single roots and those with double roots were similar.

**Table 5:** Evaluation of the relationships of various parameters with the amount of bone loss

		Amount of bone loss								Chi Square Test	
		0		< 25%		25–50%		> 50%			
		*n*	%	*n*	%	*n*	%	*n*	%	X^2^	*p*
**Sex**	Female	65	55.1	24	20.3	23	19.5	6	5.1	**12.242**	**0.007**
	Male	27	32.9	25	30.5	18	22	12	14.6		
**Tooth**	Right premolar	48	48	22	22	21	21	9	9	**0.709**	**0.871**
	Left premolar	44	44	27	27	20	20	9	9		
**Presence of concavity**	Absent	0	0	0	0	0	0	0	0		
	Present	92	46	49	24.5	41	20.5	18	9		
**Origin of the concavity**	Cervical third of the crown	36	50.7	13	18.3	16	22.5	6	8.5	**7.950**	**0.539**
	Cervical third of the root	50	42.7	32	27.4	23	19.7	12	10.3		
	Middle third of the root	4	57.1	1	14.3	2	28.6	0	0		
	Apical third of the root	0	0	0	0	0	0	0	0		
	Middle third of the crown	2	40	3	60	0	0	0	0		
**Amount of bone loss on panoramic radiograph**	0	89	89.9	10	10.1	0	0	0	0	**325.381**	**0**
	< 25%	3	6.5	33	71.7	10	21.7	0	0		
	25–50%	0	0	6	15	29	72.5	5	12.5		
	> 50%	0	0	0	0	2	13.3	13	86.7		
**Location of furcation**	0	45	48.4	23	24.7	21	22.6	4	4.3	**25.215**	**0.003**
	Cervical Third	0	0	0	0	0	0	2	100		
	Middle Third	16	38.1	11	26.2	9	21.4	6	14.3		
	Apical Third	31	49.2	15	23.8	11	17.5	6	9.5		
**Number of roots**	Single	45	48.4	23	24.7	21	22.6	4	4.3	**4.185**	**0.242**
	Double	47	44.3	26	24.5	20	18.9	13	12.3		

*n*, number; X^2^,chi-square; *p*, *p* value

Table 6 shows that age had a significant impact on the amount of bone loss (F = 14.458, *p* = 0.000), indicating that as bone loss increased, the mean age also increased. For example, while the mean age in the group with no bone loss was 39.25 years, this mean value increased to 49.67 years in the group with more than 50% bone loss.

The concavity depth had a significant relationship with the amount of bone loss (F = 5.834, *p* = 0.001), indicating that as bone loss increased, the mean depth also increased. In particular, the mean depth was greatest at 1.29 mm in the group with more than 50% bone loss.

Other measurements, such as buccolingual length, length in the crown and root according to the cementoenamel junction, apicocoronal length, buccal and palatal root length, and buccopalatinal cervical root thickness, did not significantly influence the amount of bone loss (p values: 0.542, 0.990, 0.827, 0.876, 0.628, 0.360 and 0.474, respectively).

These results demonstrated that age and concavity depth had significant relationships with the amount of bone loss. Other anatomical measurements did not show a direct relationship with bone loss in this study.

**Table 6:** Effect of variables on the amount of bone loss for upper premolar teeth

	Amount of bone loss												ANOVA	
	0			< 25%			25–50%			> 50%				
	Mean	SD	M	Mean	SD	M	Mean	SD	M	Mean	SD	M	F	*p*
**Age**	39.25	12.23	38	51.47	12.31	55	49.15	10.05	50	49.67	13.28	54	**14.458**	**0**
**Concavity depth (mm)**	0.94	0.35	1	0.95	0.39	0.89	0.89	0.3	0.82	1.29	0.4	1.18	**5.834**	**0.001**
**Buccolingual length (mm)**	4.25	0.87	4.21	4.33	0.97	4.24	4.37	0.71	4.22	4.57	1.14	5.04	**0.719**	**0.542**
**Distance from CEJ on the crown (mm)**	0.55	0.8	0	0.53	0.96	0	0.57	0.73	0	0.49	0.77	0	**0.038**	**0.99**
**Distance from CEJ on the root (mm)**	7.1	1.95	7	7.44	2.13	7.41	7.18	2.16	7.4	7.17	2.26	7	**0.298**	**0.827**
**Apicocoronal length (mm)**	7.65	2.21	7.6	7.98	2.26	8	7.75	2.35	8.2	7.67	2.36	7.34	**0.229**	**0.876**
**Buccal root length**	13.48	1.93	13.74	13.64	1.3	13.61	13.17	1.46	13.32	13.49	2.05	14.01	**0.582**	**0.628**
**Palatinal root length**	12.64	1.67	13.01	12.93	1.55	12.91	12.29	1.65	12.01	12.02	2.13	12.31	**1.082**	**0.36**
**Buccopalatinal cervical root width (mm)**	8.29	0.68	8.4	8.27	0.77	8.4	8.13	0.81	8.22	8.06	0.73	8.1	**0.838**	**0.474**

SD, standard deviation; M, median; F value; *p*, *p* value

## Discussion

In this investigation, CBCT-generated data on mesial root concavity characteristics, the location of the furcation and the length of the roots of the upper first premolar teeth were analysed in relation to periodontal status. The present study demonstrated that the apicocoronal length, the depth of the concavity and the location of the furcation relevant to the CEJ in maxillary first premolars with mesial root concavity varied between individuals. These variations increase the risk for periodontal bone loss by favouring biofilm deposition and negatively impact debridement efficacy.

There was mesial concavity in the upper first premolar teeth in the entire population examined in our study. A recent study performed by Chen et al. analysed a total number of 343 maxillary premolars (167 teeth from the right side and 176 from the left) and they found the prevelance of mesial concavity 62.5% (*n* = 110) for the left and 68.9% (*n* = 115) for the right maxillary first premolars [13]. A previous CBCT study performed by Fan et al. revealed that 64.5% of single-root maxillary premolars had mesial cervical concavity, while 73.8% of two-root maxillary first premolars had root concavity [5]. The reason for this discrepancy in the distribution of the first premolar root concavity may be attributable to differences in race and geographical region. Another CBCT investigation by Zhao et al. revealed that the concavity rate was 100% among their samples [11]. Variations in CBCT equipment, exposure parameters, and measurement techniques can also lead to disparate study outcomes. In a study on extracted teeth, concavity was observed at a rate of 100% among 50 samples, as we found in our investigation [14]. Although the methods used in the studies vary, it can be assumed that root concavity has been observed at high rates based on the results of these studies [11, 14].

In our study, among the 200 teeth evaluated, 71 had concavity in the crown cervical region, 117 in the root cervical region, 7 in the middle third of the root, and 5 in the middle third of the crown. These types of root concavities may promote the retention of bacterial plaque and generate an environment that is conducive to the development of periodontal disease [6, 15].

In the present study, 46.6% of the 200 teeth were single-rooted, and 53.4% were double-rooted. In a study by Bulut et al., 28.2% of maxillary first premolar teeth had one root, while the majority (70.8%) had two roots in the Turkish population [16]. According to a study by Kocani et al., 70.14% (*n* = 155) had two roots, 21.72% (*n* = 48) had one root, and 8.14% (*n* = 18) had three roots [17]. The sample size and inclusion criteria of the two studies could be the cause of this discrepancy. Upper premolars with a contact gap greater than 1 mm relative to the canine were excluded from our analysis.

Our study revealed that the furcation area of the 107 double-rooted teeth was 2 in the cervical third, 42 in the middle third, and 63 in the apical third. The outcomes of our research were consistent with the results of Joseph et al.’s study on extracted teeth, which revealed that the furcation site was mostly in the middle and apical third [18]. Our investigation showed that alveolar bone loss was significantly impacted by furcation location (X2 = 25.215, *p* = 0.003). In 100% of the patients with furcation in the cervical third and 9.5% in the apical third, there was more than 50% bone loss. Based on microbial adhesion and cleaning challenges, this finding has indicated that furcation may hasten disease progression as it approaches the crown [6]. This is an important finding that is difficult to obtain from conventional dental radiographs.

The amount of bone loss and the concavity depth were significantly correlated (F = 5.834, *p* < 0.001). Bone loss increased with concavity depth in the group with more than 50% bone loss, and the mean depth exhibited the greatest value of 1.29 mm. This outcome could be explained by the lack of cleanable areas at greater depths, which creates an ideal habitat for the growth of pathogens that cause periodontal disease. The depth of the concavity is a particular concern for the debridement of root surfaces even when surgical approaches are utilized [13].

The goals of treating periodontitis include stopping the disease’s progression, reducing symptoms and how they are perceived, potentially restoring lost tissue, and assisting patients in maintaining their periodontium health [19]. Various therapeutic interventions are used in periodontal treatment to accomplish these goals. These interventions include behavioural-change strategies such as customized oral hygiene instructions, support in quitting smoking, dietary modification, subgingival instrumentation to remove calculus and plaque, systemic and local pharmacotherapy, and different kinds of surgery. Combining therapy techniques with a lifetime commitment to periodontal self-care is necessary for the management of periodontal disease [20]. According to our research, the presence of mesial concavity in the upper first premolar teeth could be a factor in the development of periodontitis. In this instance, treatment should focus on these unique anatomical regions with appropriate instruments, such as Mini Five curettes, whose blades are half the size of After Five or regular Gracey curettes [21]. A shorter blade allows easier insertion and adaptation in deep, narrow pockets; furcation areas; developmental grooves; line angles; and deep, tight facial, lingual, or palatal pockets [22]. In any area where root morphology or tight tissue prevents full insertion of the standard Gracey or After Five blade, Mini Five curettes can be used with vertical strokes, with reduced tissue distention and no tissue trauma [23].

Both CBCT and panoramic images were utilized in our investigation to assess alveolar bone loss. As a result, some samples that showed less than 25% bone loss on CBCT did not appear to show any alveolar bone loss on panoramic X-ray examination. It is likely that two-dimensional imaging does not reveal the shape of the proximal alveolar bone or subgingival tissues enough. Because of canine and premolar contact, concavity may not be observed in panoramic images. Considering that panoramic radiographs may exhibit rotational projection and superposition, CBCT can be assumed to be a more reliable method than panoramic radiography. Furthermore, CBCT might be a more practical and beneficial imaging method for evaluating periodontal bone abnormalities [24]. To assist in periodontal diagnosis and treatment planning, it is important to establish selection criteria that specify the conditions and particular indications for the use of CBCT in periodontology.

The absence of a clinical assessment and not excluding other local and systemic factors that might be associated with periodontal destruction were two potential limitations of the present study. Further studies with a prospective design supported by clinical data are needed to more clearly reveal the relationship between root concavity and periodontal destruction.

## Conclusions

According to the outcomes of this study, the depth of the mesial root concavity and the coronal position of the furcation may increase the amount of alveolar bone loss. CBCT image evaluation would be beneficial to clinicians in identifying root concavity variations and selecting appropriate techniques to perform periodontal treatment successfully.

## Data Availability

The datasets used and/or analysed during the current study are available from the corresponding author upon reasonable request.

## Abbrevations

BL: Bone loss
CBCT: Cone beam computed tomography
CEJ: Cemento enamel junction
2D: Two dimensional
ICC: Intraclass correlation coefficent
n: Number
M: Median
X^2^: Chi square
F: F value
p: P value
